# Supplementary material for: A Starch- and Sucrose-Reduced Diet in Irritable Bowel Syndrome Leads to Lower Circulating Levels of PAI-1 and Visfatin: A Randomized Controlled Study
Source: Nutrients. 2022 Apr 19;14(9):1688. doi: 10.3390/nu14091688 (PMC9101041; doi:10.3390/nu14091688)
Supplement: Supplementary file 1 [file nutrients-14-01688-s001.zip › nutrients-1636282-supplementary.pdf]

**Methods of hormonal analyses**

Measurement of serum CCK was carried out by enzyme-linked immunosorbent assay (ELISA) (serial number 2D4E070E57, Cloud-Clone Corp., Oxfordshire, UK). Standard or sample of 50 μL were added to each well. Thereafter, 50 μL of detection reagent A was added to every well. The wells were shaked and incubated for 1 h at 37 °C. Afterwards, the wells were aspirated and washed 3 times with 350 μL wash solution. 100 μL detection reagent B was added to each well and thereafter incubated for 30 min in 37 °C. After incubation the aspiration and washing process was repeated 5 times. 90 μL substrate solution was then added to all the wells and incubated for 10-20 min at 37 °C. 50 μL stop solution was added to all the wells. Measurement was immediately conducted at 450 nm optical density by a microplate reader.

To measure enkephalin by ELISA (Cusabio, Fannin, Houston, USA), 100 μL standard or sample were added to each well, and thereafter the plate was incubated at 37 °C for 2 h. The liquid was removed from the wells without washing and incubation at 37 °C for 1 h with 100 μL Biotin-antibody in each well was performed. Thereafter the wells were washed 3 times with Wash Buffer. 100 μL Horseradish peroxidase (HRP)-avidin was added to each well, thereafter the plate was incubated for 1 hour at 37 °C. The washing process was repeated. 90 μL TMB substrate was added to each well, which was incubated for 20 min at room temperature (RT). 50 μL stop solution was added and measurement was immediately conducted at 450 nm optical density by a microplate reader.

Plasminogen activator inhibitor-1 (PAI-1) was measured by ELISA (Thermo Fisher Scientific, Waltham, Massachusetts, USA). The microwell stripes were washed and aspired twice with wash buffer in each well. 100 μL assay buffer was then added to the standard wells and the blank wells. Thereafter 100 μL prepared standard was added to the first standard well, which was mixed and transferred to each standard well. 50 μL assay buffer and 50 μL prediluted sample was added to each sample well. 50 μL Biotin-conjugate was added to each well and incubated for 2 h at RT. Thereafter the microwell stripes were washed 3 times. 100 μL diluted Streptavidin-HRP was added to each well and incubated at RT for 1 h. The microwell stripes were washed 3 times. TMB substrate was added to all the wells, which was incubated for 10 min at RT. Stop solution was added to each well and reading was instantly conducted at 450 nm with the help of a microplate reader.

The Mesoscale Discovery® (MSD, Maryland, USA) R-PLEX® singelplex or multiplex assay and U-PLEX® singelplex assay metabolic group (Human) were used to perform the analyses of Visfatin, NPY, and Grehlin (total and active), respectively, by electro-chemiluminescence detection [38]. The intensity of emitted light is proportional to the amount of Visfatin, NPY or Grehlin in the wells.

Biotinylated Grehlin capture antibody, 25 μL /well, was added to MSD GOLD™ small spot streptavidine plate and incubated overnight at 4°C. A metabolic assay working solution (MWS) containing aprotinin, DPP-IV inhibitor (DPP4, Merck, Darmstadt, Germany) and diluent for dilution of calibrator and samples of Grehlin active and aprotonin in diluent for Grehlin total was prepared for dilution of calibrator and EDTA plasma sample. Calibrator (active 9 070 – 2.2 pg/mL, total 5 180 – 1.3 pg/mL) and EDTA plasma (1:2 in each diluent), 50 μL /well, were added after the plates been washed three times with MSD wash buffer. A 2-hour incubation in RT was followed by a new washing procedure and a SULFO-TAG™ detection antibody, 50 ul/well, was added. After a 1-hour incubation and a washing procedure, 150 μL MSD GOLD™ read buffer B in each well was added and the plates were read on a MSD instrument.

Biotinylated NPY capture antibody was coupled with a linker, and 50 μL of the antibody solution was added to each well on a U-PLEX® plate and incubated overnight at 4°C. Calibrator (500 000 – 122 pg/mL) and serum sample, diluted 1:2, 25 μL/well, were added after the plates been washed three times with MSD wash buffer. A 1-hour incubation in RT was followed by a new washing procedure and a SULFO-TAG™ detection antibody, 50 μL /well, was added. After a new 1-hour incubation and a washing procedure, 150 μL MSD GOLD™ read buffer A in each well was added and the plates were read on a MSD instrument.

Biotinylated Visfatin capture antibody, 25 μL/well, was added to MSD GOLD™ small spot streptavidine plate and incubated overnight at 4°C. Calibrator (2 500 – 0.61 ng/mL) and serum sample,

diluted 1:2, 25 μL /well, were added after the plates been washed three times with MSD wash buffer. A 1-hour incubation in RT was followed by a new washing procedure and a SULFO-TAG™ detection antibody, 50 μL/well, was added. After a new 1-hour incubation and a washing procedure, 150 μL MSD GOLD™ read buffer in each well was added and the plates were read on a MSD instrument.
